# A Novel KIDINS220 Pathogenic Variant Associated with the Syndromic Spastic Paraplegia SINO: An Expansion of the Brain Malformation Spectrum and a Literature Review

**DOI:** 10.3390/genes15091190

**Published:** 2024-09-10

**Authors:** Maria Teresa Bonati, Cristina Baldoli, Jacopo Taurino, Daniela Marchetti, Lidia Larizza, Palma Finelli, Maria Iascone

**Affiliations:** 1 Institute for Maternal and Child Health “Burlo Garofolo”, 34137 Trieste, TS, Italy; 2 Department of Neuroradiology, San Raffaele Scientific Institute, 20132 Milan, MI, Italy; baldoli.cristina@hsr.it; 3 Cardiovascular-Genetic Center, IRCCS Policlinico San Donato, 20097 San Donato Milanese, MI, Italy; jacopo.taurino@grupposandonato.it; 4 Laboratorio di Genetica Medica, Azienda Socio Sanitaria Territoriale Papa Giovanni XXIII, 24127 Bergamo, BG, Italy; dmarchetti@asst-pg23.it (D.M.); miascone@asst-pg23.it (M.I.); 5 Research Laboratory of Medical Cytogenetics and Molecular Genetics, IRCCS Istituto Auxologico Italiano, 20095 Cusano Milanino, MI, Italy; l.larizza@auxologico.it; 6 SC Patologia Clinica, SS Laboratorio Genetica Medica, Fondazione IRCCS Ca’ Granda Ospedale Maggiore Policlinico, 20122 Milano, MI, Italy; palma.finelli@unimi.it; 7 Department of Medical Biotechnology and Translational Medicine, University of Milan, 20122 Milan, MI, Italy

**Keywords:** spastic paraplegia, macrocephaly, obesity, scaffold protein, axon guidance defects, tubulin-related disorders

## Abstract

Background/Objectives: Identifying novel variants in very rare disease genes can be challenging when patients exhibit a complex phenotype that expands the one described, and we provide such an example here. A few terminal truncating variants in *KIDINS220* cause spastic paraplegia (SP), intellectual disability (ID), nystagmus, and obesity (SINO, MIM #617296). Prompted by the result of next-generation sequencing on a patient referred for SP associated with complex brain dysmorphisms, we reviewed the phenotype of SINO patients focusing on their brain malformations, mainly described in prenatal age and first years of life, and tried to understand if the predicted effect of the mutant kidins220 may have caused them. Methods: We performed whole exome sequencing (WES) and a literature and mutation databases review. Results: We report a young adult with SP, severe ID, strabismus, and macrocephaly exhibiting brain malformations at follow-up, partially overlapping with those described in *TUBB3* tubulinopathy. WES analysis of the proband and parents identified the heterozygous de novo variant (NM_020738.4: c. 4144G > T) p. Glu 1382* in *KIDINS220* that was predicted to be causative of SINO. Conclusions: The progression of myelination and the development of brain structures turned out to be crucial for identifying, at follow-up, the whole *KIDINS220*-related brain malformations. The truncated proteins associated with SINO lack a portion fundamental for the interaction of kidins220 with tubulins and microtubule-associated proteins. The complexity of the brain malformations displayed by our patient, and possibly by other reported SINO patients, could result from an impaired dynamic modulation of the microtubule cytoskeleton during embryogenesis. Brain malformations must be considered as part of the SINO spectrum phenotype.

## 1. Introduction

*KIDINS220* (kinase D-interacting substrate of 220-kDa) encodes an ankyrin repeat-rich membrane-spanning (ARMS) protein that is required for neuronal differentiation [1,2,3,4,5].

Four heterozygous *KIDINS220* pathogenic variants have been reported in as many sporadic patients affected by spastic paraplegia (SP), intellectual disability (ID), and obesity [6,7]. Three of these patients also exhibited nystagmus and squint, as well as macrocephaly and tall stature [6]. The acronym SINO for SP, ID, nystagmus, and obesity (MIM #617296) has been created for this new syndromic SP. Autosomal dominant inheritance has been documented through a family in which the mother and both her sons exhibited the complete SINO syndrome spectrum phenotype [8]. A further heterozygous *KIDINS220* pathogenic variant has been found to segregate in a family affected by autosomal dominant pure SP [9], representing the mildest end of the SINO spectrum.

Homozygous *KIDINS220* pathogenic variants inherited from healthy parents were reported to be lethal in fetuses due to either severe progressive hydrocephalus within the twentieth gestational week [10,11] or congenital heart disease and hydrops fetalis [12]. Since the fetuses had contractures involving all limbs, their phenotype could be considered to be at the more severe end of the SINO spectrum. Lastly, only one compound heterozygous SINO patient exhibited a phenotype whose severity could be between the prenatal cases and the heterozygous patients [13].

We report on an additional sporadic patient found to be heterozygous for the novel nonsense variant *KIDINS220* (NM_020738.4): c. 4144G>T, p. (Glu 1382*) who exhibited a pattern of brain malformations, partially overlapping with those described in tubulinopathies. Therefore, we reviewed brain malformations *KIDINS220* related and aimed to understand whether they could be considered part of the SINO spectrum phenotype. We concluded that the repetition of neuroimaging should be considered in the follow-up of SINO patients to identify all brain malformations, which could result from impaired interaction, in neuronal cells, of truncated kidins220 with tubulins and microtubule-associated proteins damaging the dynamic modulation of the microtubule cytoskeleton, assembled during embryonic development from different α- and β-tubulin isotype heterodimers. Moreover, we suggest considering *KIDIN220* in diagnosing complex brain malformations.

## 2. Materials and Methods

### 2.1. Subjects

Clinical and instrumental evaluation and genetic testing were accomplished as part of routine clinical care. The patient’s parents signed informed consent for genetic analyses, clinical evaluation, and publication.

### 2.2. Brain Imaging

Brain Magnetic Resonance Imaging (MRI) examinations were performed under sedation on a Philips Achieva 1.5 T scanner using an 8-channel Sense Head coil; a neuroradiologist reviewed them with pediatric expertise. The scans consisted of TSE T2 multiplanar images, T1 SE axial and sagittal images, volumetric 1 mm thick T1 images, DWI and DTI (21 directions) studies, and FLAIR axial images 3 and 4 mm thick.

### 2.3. Genetic Analysis

Genomic DNA was extracted from the peripheral blood samples of proband and parents using standard procedures. The genome’s exonic regions and flanking splice junctions were captured using the Clinical Research Exome *v*.2 kit (Agilent Technologies, Santa Clara, CA, USA). Sequencing was performed on a NextSeq500 Illumina system with 150 bp paired-end reads. Reads were aligned to human genome build GRCh37/UCSC hg 19 and analyzed for sequence variants using a custom-developed analysis tool [14]. Additional sequencing technology and variant interpretation protocols have been previously described [14]. Coverage on target for the index was ≥10× for 98.3% with a mean coverage of 214×.

### 2.4 Literature Review

We employed databases of DNA variants, such as ClinVar [15], HGMD^®^ Professional [16], and PubMed [17], to identify patients affected by SINO. We reviewed all the clinical aspects of these patients and summarized the phenotype and genetic data in a table shown in the main text.

Then, we compared the merged phenotype of prenatal cases and postnatal patients affected by SINO with the phenotype of patients with *TUBB3* tubulinopathy and the patient described here. We reported the comparison in a supplementary table.

## 3. Results

### 3.1. Clinical Report

The patient was the firstborn to healthy unrelated, Caucasian parents, delivered after 38 weeks and 5 days of gestation by C-sec because of breech presentation. Their family history was unremarkable. The pregnancy was characterized by the risk of abortion in the second month, treated with vasosuprine, and by the finding of unilateral ventriculomegaly on ultrasound (US) performed in the third trimester. His birth weight was 3080 g (33rd centile), birth length 51 cm (77th centile), and occipitofrontal circumference (OFC) 35.5 cm (85th centile). His Apgar scores were 8 and 9 at 1 and 5 min. Transfontanellar US of the neonatal brain did not confirm the malformation.

The patient underwent his first neurological examination for motor milestones delay at 12 months when the adductive hyper-tone of the lower limbs was noticed. He sat alone and stood with help at 15 and 19 months, respectively, and began to walk assisted at 22 months. At this age, he was diagnosed with psychomotor delay, so he underwent audiometry, ophthalmologic examination, EEG recording, and ECG, which revealed normal findings. He was able to say his first words at around 24 months. At 7 and a half years old, he could understand simple sentences and express his needs despite dislalia and letter omissions. He tended to stay isolated from peers, be oppositional towards any new proposals, and have tantrums, as well as severe attention deficit, temporal organization, and graphic representation. At 12 years, his cognitive function was measured by WISC-III to be severely delayed, with a total IQ equal to 32 and verbal and performance IQ equal to 43 and 36, respectively. From his clinical records, we could determine that at 21 and 28 months, his OCF was >97th and weight between 50 and 75th centile, whereas the length curve moved from the 3rd to the 10th centile. At the age of 15 years and 10 months, while on a diet, his weight decreased from 81 (>90th centile) to 70 kg (just >75th centile).

For spastic paraplegia, he underwent orthopedic surgery several times from the age of 4 years to 11. For the bladder sphincter control, he had been toilet trained since his first years of life, whereas he always needed microclisms to evacuate the stool. Cardiac US at 17 years was average.

**Figure 1:**
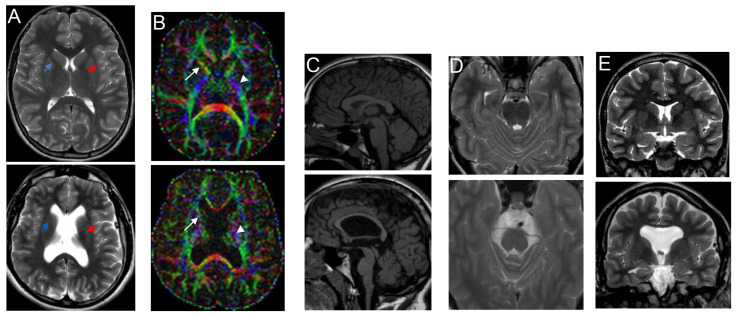
Brain MRI scans of the 17-year-old patient (bottom panel) compared to an age-matched control (upper panel): (**A**) transverse T2 SE, (**B**) color fractional anisotropy, (**C**) sagittal T1 SE, (**D**) transverse T2 SE, (**E**) coronal T2 SE. (**A**) Lateral ventricle enlargement with dysmorphic appearance; the dysmorphic and hypoplasic anterior limb of the internal capsule (light blue arrow) with partial fusion of lenticular nucleus and caudate nucleus head (red arrow). (**B**) Thinning of the corticospinal tract is evident at the level of the posterior arm of the internal capsule (white head arrow). (**C**) Thinned and dysplasic corpus callosum. (**C**,**D**) Pontomesemcephalic hypoplasia with defect of segmentation and dysmorphic appearance. (**E**) Septum pellucidum agenesis, thickened and dysmorphic fornices, anterior commissure agenesis, optic chiasm hypoplasia. (**A**,**E**) Simplified pattern of gyration of the frontal lobes with global volumetric reduction.

Brain MRI performed at the age of 17 years displayed a pattern of malformative dysmorphisms, especially involving the brainstem, which exhibited hypoplasia and dysplastic appearance of the pontomesencephalic junction (Figure 1C,D) and the supratentorial midline structures with communication between lateral ventricles anteriorly, comprehensive communication of lateral ventricles with the third ventricle, partial agenesis of pellucidum septum, corpus callosum hypoplasia, coarseness/fusion of fornices, and verticalized hippocampi (Figure 1A,C,E).

These features were all documented at 22 months. In contrast, at 17 years, there was also evidence of poor bilateral delimitation of the anterior arm of the internal capsule with apparent partial fusion of lenticular nucleus with nucleus caudate head (Figure 1A), thinned and dysmorphic corticospinal bundle (Figure 1B), the absence of superior cerebellar peduncles decussation (evidenced by DTI study), simplification of the cortical gyration pattern, and symmetrical reduction in frontal and temporal lobe volume. A slight thinning of the optic chiasm and nerves was evident (Figure 1A,E). EEG still did not detect abnormal findings.

As neuroradiological findings were suggestive of TUBB3 tubulinopathy, the patient was referred for genetic evaluation. At the age of 18 years, his height was 178 cm~ (50–75th centile), weight 76 kg (75th centile), and OFC 59 cm (>97th centile). He exhibited slight turricephaly, divergent strabismus of the right eye, accentuation of kyphosis, semi-flexed hips, and flexion of the knees. The presence of frequent stereotyped movements with his head (shaking it as if to say ‘no’) and with his hands (such as clapping, slapping surfaces, his parents, and his head, and hand flapping), as well as verbal language not addressed to others, tantrums, and short attention, were all indicators of poor cognitive functioning, as measured by WISC-III at the age of 12 years. He could walk with the support of articulated braces or tripods, but he used a wheelchair outdoors. He was attending educational therapy to improve the use of verbal communication.

Routine chromosomal blood analysis presented a normal male karyotype; fragile X syndrome was ruled out before the second pregnancy of his parents. Array Comparative Genomic Hybridization analysis performed on genomic blood DNA using the SurePrint G3 Human CGH Microarray Kit 2x400K did not detect copy number variants (CNVs). Growth parameters did not match the clinical diagnosis of tubulinopathy (Table S1); therefore, we proposed a trio-WES analysis (instead of a panel sequencing approach).

### 3.2. Genetic Findings

**Figure 2:**
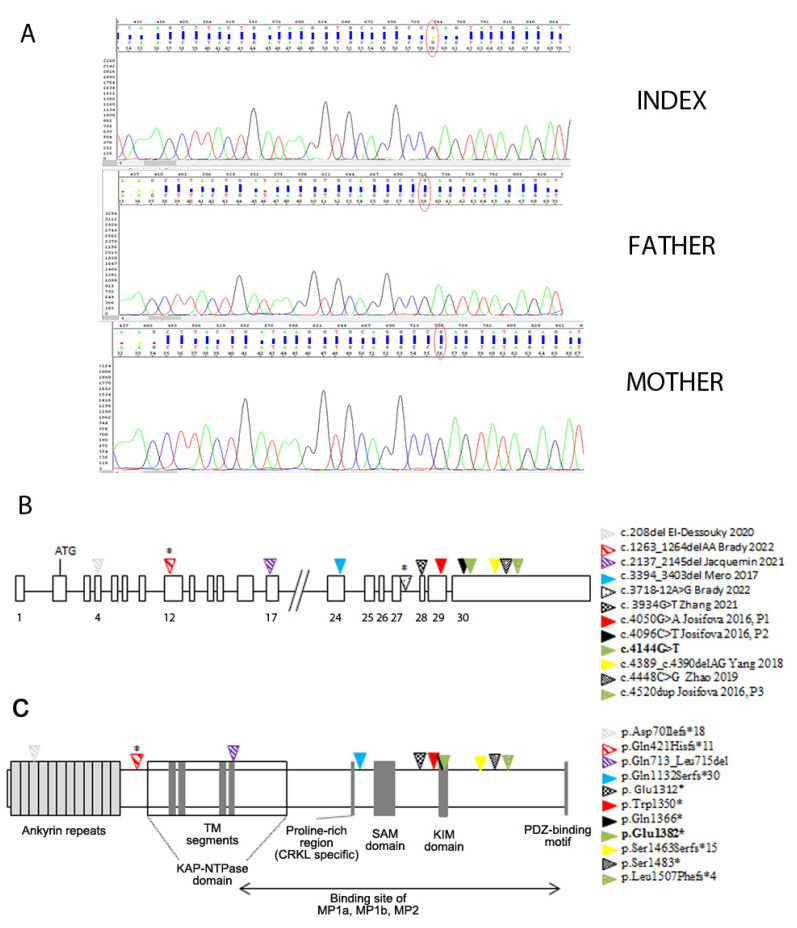
*KIDINS220* pathogenic variants associated with SINO. (**A**) Electropherogram showing the de novo nonsense heterozygous variant. (**B**) The genomic structure of *KIDINS220* includes 30 exons. The approximate position of the pathogenic variants, based on RefSeq NM_020738.4, is indicated; the novel variant reported here is highlighted in bold. (**C**) Schematic structure of Kidins220/ARMS with its six putative domains: I, ankyrin repeats domain; II, KAP (kidins220/ARMS and PifA)-NTPase domain; III, proline-rich domain: the region crucial for docking the CRKL adaptor protein is necessary for the sustained activation of MAPK signaling; IV, SAM (sterile α motif) domain; V, KIM (KLC-interacting motif) domain; KLC, kinesin light chain; VI, PDZ (PSD-95, Dlg, and ZO-1 protein)-binding motif domain. Domains III to VI constitute the C-terminal domain. Triangles show the approximate position of the eleven pathogenic variants in the protein domains. The impact of the predicted splice variant in intron 27 has yet to be studied, so it is not reported. P, patient; TM, transmembrane; *, variants in compound heterozygosity. References: Josifova 2016 [6], Yang 2018 [7], Zhang 2021 [8], Zhao 2019 [9], Mero 2017 [10], Jacquemin 2021 [11], El-Dessouky 2020 [12], Brady 2022 [13].

The WES analysis of the proband and both parents identified a novel de novo KIDINS220 variant in exon 30, Chr2 (GRCh37): g.8872022 (NM_020738.4: c.4144G > T) (Figure 2A), resulting in a premature stop codon, predicted to lead to the truncated protein p. (Glu 1382*).

No rare variants were identified in genes involved in spastic paraplegia.

An overview of the *KIDINS220* pathogenic variants associated with SINO is shown in Figure 2. Apart from the splicing variant, all the others (five nonsense and six frameshifts due to five small deletions and one small insertion) were predicted to lead to truncated proteins. As can be seen in Figure 2B, the variants detected in homozygosity in the aborted fetuses [10,11,12] had an upstream location (exons 4, 17, and 24), whereas all the others, which cluster in exons 28–29 and mainly in the last exon 30, were found in heterozygosis. The variants located downstream the position at ~55 bp upstream of the last exon–exon junction were predicted to escape nonsense-mediated mRNA decay (NMD) [10] (Figure 1).

The SINO-related variants are listed in the first column of Table 1, together with data on familial transmission and the phenotype of patients/prenatal cases, with *a focus on* brain malformations.

### 3.3. Spectrum of Brain Malformation in Prenatal Cases and Postnatal Patients Affected by SINO

As shown in Table 1, prenatal US data were available for seven postnatal patients out of fourteen; nine prenatal cases completed the series. Out of twenty-three cases, seven were unrelated probands affected by SINO, and there were three triplets of unborn sibling fetuses. Dilated ventricles and thinning/agenesis of the corpus callosum were present in almost all the series. In addition, cerebellar involvement was identified in two prenatal cases. In contrast, more complex dysmorphisms, detailed in the discussion section, were identified in postnatal life, as expected by the progression of myelination and development of brain structures (patients’ age at brain MRI is specified in Table 1).

Recurrent craniofacial dysmorphisms, also in prenatal cases, included brachycephaly and plagiocephaly, a bossed/high/prominent forehead, and micrognathia.

Lastly, Table S1 shows the overlap of the clinical features between the reported SINO series [6,7,8,9,10,11,12,13] and the TUBB3-related spectrum [20,21,22,23,24,25,26,27,28]. It is noteworthy that, with the exception of growth parameters, there is a wide overlap between SINO and the TUBB3-related spectrum in terms of developmental and neurological and ophthalmological impairments, with an invariable presence of SP in SINO. Moreover, the comparison with our patient allowed us to conclude that he widened the spectrum of the brain dysmorphisms associated with SINO, as discussed later.

## 4. Discussion

We report on the second adult affected by syndromic spastic paraplegia SINO, but the first one whose brain malformations widen those previously described in five young patients (ages at neuroimaging between 12 and 36 months) [6,7,13] and unborn sibling fetuses [10,11,12] (Table 1). Indeed, hypoplasia of the corpus callosum (Figure 1C) and enlarged, dysmorphic ventricles (Figure 1A), features already reported in SINO (Table 1), were found in our patient when followed-up at 17 years to be associated with a simplified gyration pattern (Figure 1A,E), poor delimitation of the anterior limb of the internal capsule (ALIC), partial fusion of the lenticular nucleus with the nucleus caudate head (Figure 1A), a thinned and dysmorphic corticospinal bundle (Figure 1B), the absence of superior cerebellar peduncles decussation, and thinning of the corticospinal tracts and optic chiasm. Agenesis/hypoplasia of ALIC and dysmorphic/small/large/fusion/unusual orientation of basal ganglia represent the hallmarks of tubulinopathies. In particular, the finding of these complex brain malformations led us to hypothesize a mutation in the *TUBB3* gene, encoding β-tubulin isotype III [20,21,22,23,24,25] (OMIM #614039) (Table S1).

Except for thinning of the corticospinal tracts and optic chiasm, the other signs of guidance axon defects were detected in our patient at the first brain MRI (22 months), i.e., at an age equivalent to that of the patients described by Josifova et al. [6] at their brain imaging (Table 1). We could not exclude that Josifova’s patients might also have exhibited some axon specification defects because their MRI scans were not shown, nor was their resolution described. Conversely, chiasma opticum and optic nerve hypoplasia were also reported in a 3-year-old girl affected by SINO [7] (Table 1) at an age when thinning of the corticospinal tracts was not yet evident. Interestingly, thin brainstem and hypoplasia of the basal ganglia and thalami were identified in the female patient with autosomal recessive SINO who underwent neuroimaging on the eighth day of life [13] (Table 1). Cerebellar involvement was detected in two prenatal cases [12] and a baby girl with bi-allelic *KIDINS220* pathogenetic variants [13], whereas lissencephaly, another feature of tubulinopathies, was reported in a further prenatal case [11]; see (Table 1).

Sporadic and autosomal dominant (AD) SINO were associated with *KIDINS220* terminal truncating pathogenic variants, whereas autosomal recessive (AR) lethal SINO had early truncating ones (Figure 2B and Table 1). It is noteworthy that in the non-lethal AR SINO, one of the two pathogenetic variants was an early truncating one that could trigger NMD, and the other was a splicing one (Figure 2), allowing the residual wild-type proteins, those correctly spliced, not to be interfered by truncated protein escaping NMD.

As a multifunctional scaffolding protein, kidins220 induces, in response to neurotrophic stimuli acting at the N-terminal domain, the sustained activation of MAPK signaling and in parallel interacts, at the C-terminal domain, with proteins that modulate the assembly and stability of microtubules with tubulins during the different phases of neuronal morphogenesis [1,2,3,4,5]. At the level of protein domains (Figure 2C), all the variants were predicted to perturb the C-terminal region. The integrity of the KIM domain, disrupted by the first eight mutations Figure 2, is important in ensuring the intracellular traffic of kidins220 to the neurite tips in response to neurotrophic stimuli [2]; the integrity of the portion between residues 760–1762, encompassing the third to the sixth domain (Figure 2C), is fundamental for the interaction of kidins220 with tubulins and the microtubule-associated proteins (MAPs) MAP1a, MAP1b, and MAP2 [3]. All the mutations shown in Figure 2, including those reported by Zhao et al. [9] in the family affected by pure SP, appear to have the comparable effect of perturbing this interaction.

Therefore, we hypothesize that the complexity of the brain malformations and the axon guidance defects displayed by the patient analyzed here, and possibly by other SINO subjects described in the literature, could depend on the impaired dynamic modulation of the microtubule cytoskeleton in neuronal cells, likely caused by a dominant-negative effect of the mutation in the heterozygotes.

Furthermore, the gene undergoes alternative splicing, which is both developmentally regulated and organ/tissue specific and consists of removing the middle region and/or the last exon. Josifova et al. [6] demonstrated that the truncated Trp1350 * and Gln1366 * proteins (Figure 2C) were similar to the alternative terminal axon (ATE) splice isoform produced in mice and humans mainly during adulthood. Strikingly, motor neurons in mice do not exhibit ATE isoforms at any time during development [29]. Therefore, the invariable presence of SP in SINO could be the result of the disrupted expression of the full-length *KIDINS220* splice isoform repertoire in motor neurons [6].

Obesity, instead, could be the result of the disruption, in the truncated kidins220 proteins, of the sustained activation of ERK signaling, which physiologically acts as a negative regulator of adipocyte differentiation and maturation [8].

Lastly, recent evidence from animal models engineered to study the pathophysiology of *KIDINS220*-linked dysfunctions involving sensory processing suggested that the impairment of auditory and olfactory responses could serve as an early biomarker for common human neuropathologies, like Alzheimer’s disease [30,31]. Furthermore, animal models helped unravel, in SINO, the pathophysiology of memory and possible behavioral phenotypes, such as anxiety and aggression [32]. We think that the results from these studies could serve as an educational setting for rare SINO patients, too.

## 5. Conclusions

In conclusion, as summarized in Table 1, SINO is a very rare syndromic SP exhibiting AR inheritance mostly in the prenatally lethal form as well as AD inheritance and de novo occurrence. Apart from the c. 4520dupT [6], identified also in a patient affected by neurodevelopmental disorder [18,19], each variant associated with SINO seems private. A broad spectrum of brain malformations, some prenatally detectable, is part of the SINO phenotype. Indeed, we hypothesize that impaired interaction of kidins220 mutants with tubulins and MAPs could determine them. The patient reported here widens the spectrum of the brain malformations/dysmorphisms, which demonstrates that accurate neuroimaging in childhood and at follow-up seems crucial in detecting the complexity of brain malformations associated with *KIDINS220* pathogenic variants.

SP instead could be due to the vulnerability of motor neurons to the kidins220 isoform lacking the terminal exon.

We provide evidence of further locus heterogeneity of cortical and brain malformations and suggest *KIDINS220* as a new candidate gene for tubulin-related disorders as well as for isolated SP and those prenatal cases that exhibit hydrocephalus and distal arthrogryposis but do not carry *L1CAM* pathogenetic variants [33].

## Figures and Tables

**Table 1:** A literature review of clinical and genetic findings of *KIDINS220*-mutated patients ordered according to the position in the gene (5’ to 3’) of the pathogenic variants.

*KIDINS220* (NM_020738.4) Variants	Inheritance	Prenatal US (WG)	Age at Brain MRI	Brain MRI	Age at Last Follow-Up Gender	Neurological/ Postmortem Examination	ID	Growth Parameters	Craniofacial Dysmorphisms	References
c. 208del; p.Asp70Ilefs*18 (exon 4)	AR (homozygous)	Dilated ventricles (3/3), CC agenesis (3/3), cerebellar hypoplasia (2/3), cerebellar vermis agenesis (2/3) ↑NT (1/3), CHD (3/3), hydrops fetalis (2/3),polyhydramnios (2/3), ascites (1/3) Normal for gestational age (1/3); NA (2/3) (12, 22 and 24)	/	NP	/ 1M, 2F	Limb contractures (3/3)	/	/	Brachyplagiocephaly (3/3), bossed forehead (3/3), deep set eyes (3/3), micrognathia (3/3)	[12]
c.1263_1264delAA p.Gln421Hisfs*11 (exon 12) pat c.3718-12A>G (inron 27) mat	AR (compound heterozygous)	Bilateral ventriculomegaly (19 +3) Further ventricle enlargement, bilateral talipes equinovarus (20 +3) Polyhydramnios (31) Large for gestational age (35 + 3)	21 and 27 WG 8th day	3rd ventricle dilatation, thin corpus callosum, possible absence of the cavum septum pellucidum Thin corpus callosum, thin brainstem, absence of the cavum septum pellucidum, hypoplasia of the basal ganglia, thalami, and inferior cerebellar vermis	2.5 y F	Coarse nystagmus SP	Severe DD At last follow-up, minimal head control, unable to sit, reach, or grab for items, vocalizations only	COF>99th p, weight at 90th p at birth COF>98th p, length<3rd p, weight at 60th p at 18 m	Frontal bossing, mild micrognathia	[13]
c.2137-2145del p.Gln713_Leu715del (exon 17)	AR (homozygous) Parents’ first-degree cousins	Severe ventriculomegaly (3/3) (14 and second-semester) Clenched hands, bent wrists, club feet (3/3) Normal for gestational age (3/3)	Post Mortem (P in 1/3)	Triventricular hydrocephalus, cortical atrophy without gyri (lissencephaly), confirmed at autopsy	/ 2M, 1F	Limb contractures (3/3)	/	/	-	[11]
c.3394_3403del; p.Gln1132Serfs*30 (exon 24)	AR (homozygous)	Hydrocephalus/dilated ventricles (2/3), CC agenesis (1/3) NA growth parameters (13, 1 Fe; 18, 2 Fe)	/	NP	/ NA	Limb contractures (3/3)	/	/	Micrognathia (1/3)	[10]
c.3934G>T; p.Glu1312* (exon 28)	AD	NA	/	NP	39 y F	Nystagmus, SP	Moderate ID (IQ 39 at WAIS)	Severe obesity (BMI 35.6 kg/m^2^)	-	[8], mother
		NA	/	NP	17 y M	Nystagmus, SP	Moderate ID (IQ 42 at WAIS)	Obesity (BMI 29.4 kg/m^2^)	Brachycephaly	[8], elder son
		Normal findings	19 m	Normal findings	5 y M	SP	Moderate ID	Early-onset overgrowth (macrocephaly, height and weight >99th p), obesity	Brachycephaly	[8], younger son
c.4050G>A; p.Trp1350* (exon 29)	de novo	Dilated lateral ventricles (23)	12 m	Dilated 3rd and lateral ventricles, ↓ WM bulk, mild delay in myelination, mild generalized atrophy	14 y M	Nystagmus, axial hypotonia, SP	Moderate ID; he speaks in sentences since 4 y	Early-onset overgrowth (OFC, height and weight >90th p)	Brachyplagiocephaly, bossed forehead, deep-set eyes	[6], patient 1
c.4096C>T; p.Gln1366* (exon 30)	de novo	Dilated lateral ventricles (20)	24 m	Dilated 3rd and lateral ventricles, ↓ WM bulk, mild generalized atrophy	15 y M	Nystagmus, squint, SP	Moderate ID; he speaks in sentences since 4 y	Early-onset overgrowth (OFC, height and weight >90th p)	Brachyplagiocephaly, prominent forehead, deep-set eyes, crowded teeth	[6], patient 2
c.4144G>T; p.Glu1382* (exon 30)	de novo	Unilateral ventriculomegaly (3rd trimester)	22 m	Lateral ventricle enlargement, pellucidum septum agenesis, CC hypoplasia, verrticalized hippocampi, thin and dysmorphic brainstem	18 y M	Monolateral squint, SP	Severe ID; he speaks in sentences	Early-onset macrocephaly, obesity	High forehead	Present study
			17 y	Also, dysmorphic and hypoplastic ALIC, partial fusion of lenticular and caudate nuclei, corticospinal tract thinning, optic chiasm hypoplasia, ↓ frontal and temporal lobe volume with cortical gyration simplification, absence of superior cerebellar peduncles decussation						
c.4389_c.4390delAG; p.Ser1463Serfs*15 (exon 30)	de novo	Normal findings	3 y	Periventricular WM high signals at FLAIR, ↓ splenium and posterior corpus callosi, chiasma opticum, and optic nerve hypoplasia	3 y F	SP	Mild ID	Obesity	Short philtrum, ectropion of nostril, plump cheeks	[7]
c.4448C>G; p.Ser1483* (exon 30)	AD	NA	/	NP (5/5)	NA 3M, 2F	SP (5/5)	- (5/5)	Normal (5/5)	- (5/5)	[9]
c.4520dup; p.Leu1507Phefs*4 (exon 30)	de novo	Dilated lateral ventricles	30m	High-riding 3rd ventricle, dilated lateral ventricles, partial CC agenesis	7 y M	Nystagmus, squint, SP	Moderate ID; few single words by 24 m	Early-onset obesity	Brachyplagiocephaly, prominent forehead	[6], patient 3; The variant has been also reported in a patient with NDD [18,19]

The gray rows show the data of aborted fetuses; WG, week of gestation; n, number; Fe, fetus; m, months; y, years; /, not applicable; +, present; -, absent; ↓, reduced; ↑, increased; P, performed; NP, not performed; NA, not available; M, male; F, female; CC, corpus callosum; WM, white matter; ALIC, anterior limb of internal capsule; ID, intellectual disability; SP, spastic paraplegia; AD, autosomal dominant; AR, autosomal recessive; mat, maternal; pat, paternal; NT, nuchal translucency; CHD, congenital heart disease; NDD, neurodevelopmental disorder

## Data Availability

Data are contained within the article or Supplementary Materials.

## Supplementary Materials

The following supporting information can be downloaded at https://www.mdpi.com/article/10.3390/genes15091190/s1, Table S1: Comparison of the comprehensive phenotype associated with mutations in *KIDINS220*, *TUBB3,* and the patient. The clinical data exhibited by each patient affected by SINO are detailed in Table 1.
