# Supplementary material for: A Novel KIDINS220 Pathogenic Variant Associated with the Syndromic Spastic Paraplegia SINO: An Expansion of the Brain Malformation Spectrum and a Literature Review
Source: Genes (Basel). 2024 Sep 10;15(9):1190. doi: 10.3390/genes15091190 (PMC11431642; doi:10.3390/genes15091190)
Supplement: Supplementary file 1 [file genes-15-01190-s001.zip › genes-3192761-supplementary.pdf]

Table S1. Comparison of the comprehensive phenotype associated with mutations in *KIDINS220*, *TUBB3* and the patient. The clinical data exhibited by each patient affected by SINO is detailed in Table 1 (main text)

	SINO	*TUBB3*-related spectrum	Present patient
**Development**			
Psychomotor developmental delay	+	+	+
Delayed speech and language development	+	+	+
Intellectual disability	+	+	+
**Neurology**			
Muscular hypotonia of the trunk	+	+ (infantile)	+
Motor impairment	(progressive) SP	spasticity, spastic ataxia	SP
Seizures	-	+	-
Axonal peripheral neuropathy	-	+	-
Other	-	feeding difficulties in infancy, impaired mastication,<br>sensorineural hearing impairment,<br>dysarthria, dystonia	-
**Brain**			
Delayed/abnormal CNS myelination	+	-	-
Reduced WM bulk/periventricular WM abnormalities	+	-	-
Ventriculomegaly/Dilation of lateral ventricles	+	+	+
Cerebral atrophy	+	-	-
Lissencephaly (type II)/ Polymicrogyria	-	+	-
Simplified gyral pattern	+	+	+
Hypoplasia/(partial) agenesis of the corpus callosum	+	+	+
Agenesis of the anterior commissure	-	+	+
Hypoplasia of internal capsule	-	+	+
Dysmorphic/small/large/fusion/unusual orientation of basal ganglia	+	+	+
Abnormality of thalamus morphology	+	+	-
Dysgenesis of the hippocampus	-	+	+ (verticalized hippocampi, as from incomplete rotation)
Cerebellar hypoplasia	+	+	+ (peduncles)
Abnormality/hypoplasia/aplasia of cerebellar vermis	+	+	-
Hypoplasia of the brainstem	+	+	+
Hypoplasia of corticospinal tracts	-	+	+
Chiasma opticum/Optic nerve hypoplasia/dysplasia	+	+	+
Hypoplastic cranial (oculomotor, facial) nerves	-	+	-
Hypoplasia of the olfactory bulb	-	+	-
Other	-	-	dysmorphic fornices, septum pellucidum agenesis, hypoplasia of brain peduncles, absence of superior cerebellar peduncles decussation, frontal and temporal lobe volume reductions

**Eye**			
Strabismus, esotropia, esophoria	+	+	+
Nystagmus	+	+	-
Oculomotor apraxia	-	+	-
Ptosis	-	+	-
**Growth**			
	Early onset overgrowth	Microcephaly in some	Early onset macrocephaly
	(Early onset) obesity	Postnatal growth retardation	Obesity

+, present; -, absent; SP, spastic paraplegia; WM, white matter
